# Supplementary material for: Generative emergence of non-local representations in the hippocampus
Source: Nat Commun. 2025 Aug 27;16:8012. doi: 10.1038/s41467-025-63346-w (PMC12391462; doi:10.1038/s41467-025-63346-w)
Supplement: Supplementary file 1 — Supplementary Information [file 41467_2025_63346_MOESM1_ESM.pdf]

SUPPLEMENTARY INFORMATION

Generative emergence of non-local representations in the hippocampus

Authors: Yuchen Zhou, Jeremie Sibille and George Dragoi

Includes:

Supplementary Figures 1-18

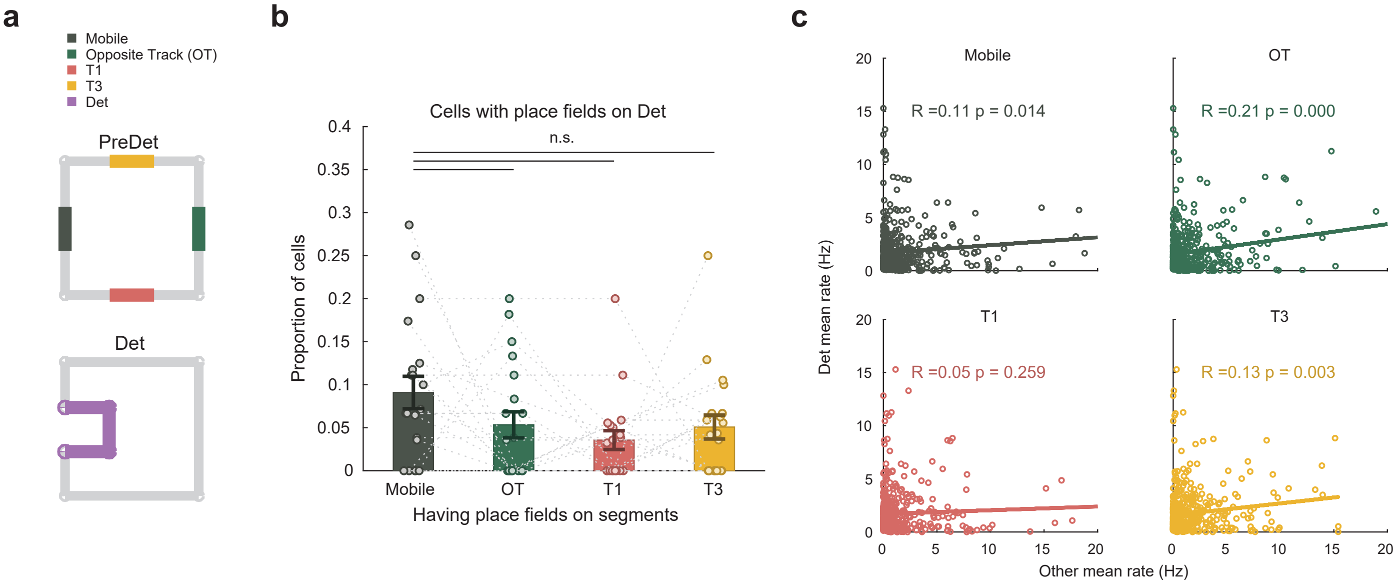

Supplementary Figure 1. Firing rate similarity between 150 cm detour segment and 50 cm middle segments from the pre-detour run session. a, Cartoon highlighting 50 cm middle segments from the pre-detour session and the 150 cm detour segments. **b**, Across cells with place fields detected on the detour segments, the ratio of cells which also have place fields with center located in the 4 middle segments in the pre-detour sessions. The ratio was not significantly higher in the pre-detour mobile segment compared to other middle segments. Each dot represents one animal, direction, and detour session (n=20). **c**, Scatter plots of cells' mean rates on detour segment and pre-detour middle segments. We observed an overall positive correlation due to the firing rates difference across cells. The correlation between the detour segment and the pre-detour mobile segment was not higher than for other middle segments. Each dot represents one cell pooled from all animals, directions, and detour sessions. n.s.=not significant.

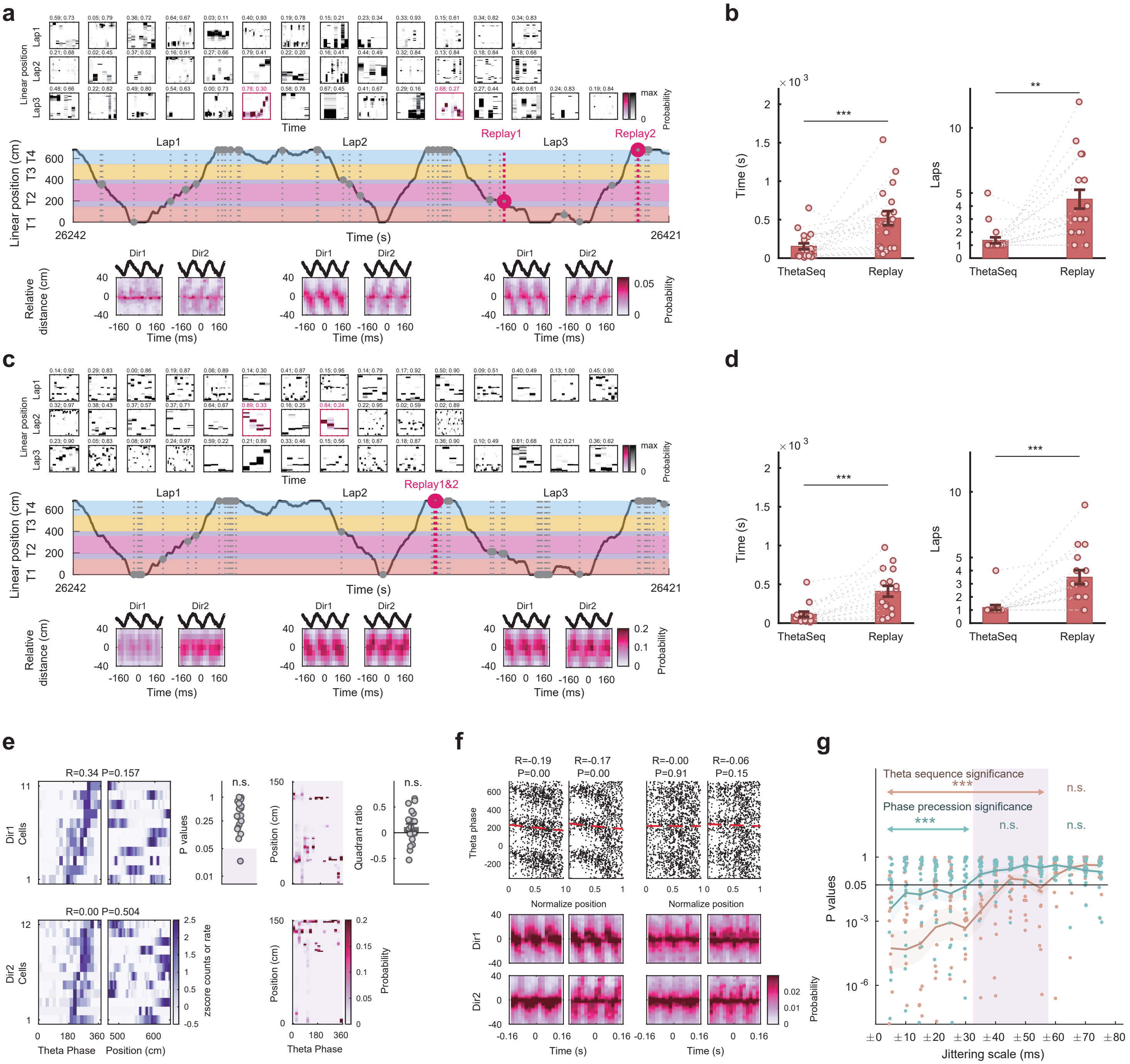

Supplementary Figure 2. Rapid expression of novel detour theta sequences earlier than replay and decorrelated from single cell theta phase mechanisms. a, Examples showing expression of detour theta sequences preceding detour waking rest replay. Top 3 rows show replay detection from all the frames with significant replays plotted in red. Subpanel titles show absolute weighted correlation and normalized maximum jump. Middle panel shows the animal's position on the maze with frames marked by dashed lines. Bottom panels show averaged theta scale decoding results in the first 3 laps. **b**, Comparison between time and lap of first significant expression of theta sequences and waking rest replay. **c-d**, Similar with (a)-(b), but with cluster-less decoding approach. **e**, Examples of first 2 laps theta phase sequences sorted by preferred spiking phase of significantly modulated cells (1st column) and their place map on the detour segment (2nd column). The correlation between theta phase sequence and place map sequence was not significant (3rd column, each dot represents one animal, direction, and detour session). The decoding results based on spike counts binned by theta phase did not exhibit any theta sequence structure (4th column) and the quadrant ratio was not significantly positive (5th column, each dot represents one animal, direction, and detour session). **f**, Examples of first 2 laps theta phase precession and theta sequence from the original data (left 2 columns) and time-jittered spikes (right 2 columns). **g**, Significance of theta phase precession and theta sequence with different levels of spike temporal jittering. Significance asterisks were plotted on top based on a binomial test against a 5% chance level, rather than comparing the mean p-value to 0.05. Data in (b, d) are represented as mean±s.e.m. with each dot representing one animal, one direction, and one detour session (n=20). Each dot in (g) represents one animal, direction, and detour session at a jittering scale. ***P<0.001, **P<0.01, n.s.=not significant.

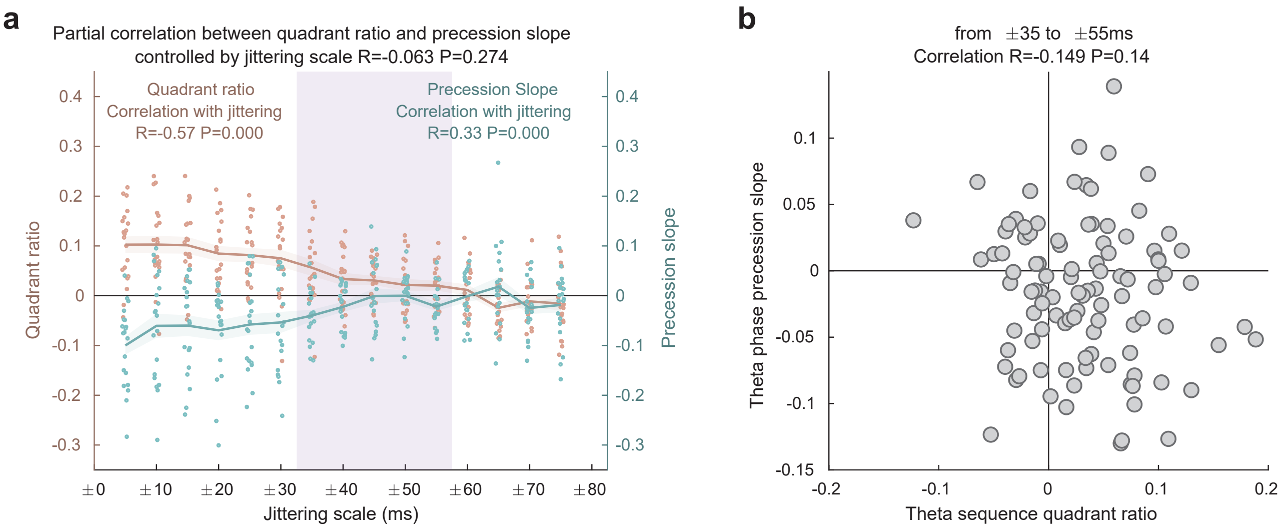

Supplementary Figure 3. Decorrelation between theta sequence and theta phase precession revealed by jittering scale. a, Plot of theta sequence quadrant ratio and theta phase precession slope across different levels of jittering. Both the quadrant ratio and precession slope were correlated with jittering scale, but the partial correlation between quadrant ratio and precession slope controlled by jittering scale was not significant. The purple area marked the range when theta sequence was significant but not the theta phase precession, based on Supplementary Figure 2g. **b**, Within the jittering scale of ±35 ms to ±55 ms, the negative correlation between quadrant ratio and phase precession slope was not significant. In this plot, each dot represents one animal, direction, and detour session at a jittering scale.

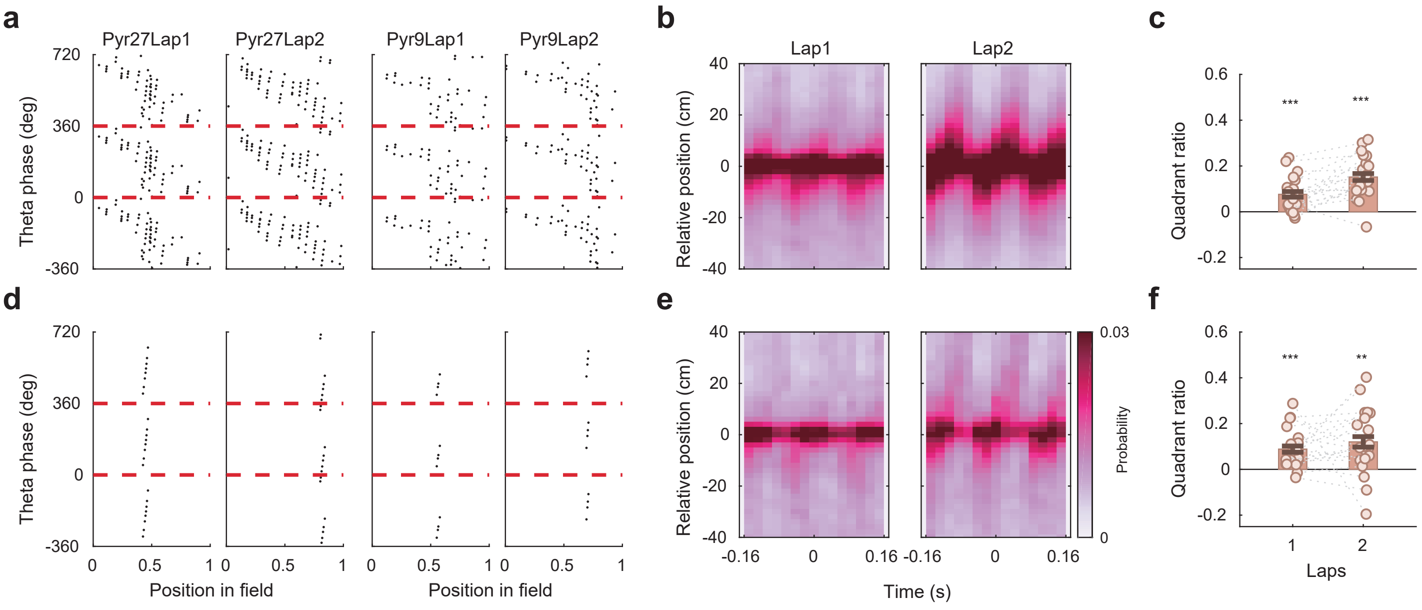

Supplementary Figure 4. Theta sequence was preserved when single cell theta phase precession was eliminated. a, Two example cells exhibiting theta phase precession on the first 2 laps on the detour segment. **b**, Decoding results of theta sequence on the first 2 laps on the detour segment averaged across all animals, directions, and detour sessions. **c**, Distribution of theta sequence quadrant ratio on the first 2 laps on the detour segment. Each dot represents one animal, direction, and detour session (n=20; Lap1, P=4.8x10-4; Lap2, P=7.6x10-5; One side Wilcoxon signed rank test with positive quadrant ratio). **d**, The same example cells from (a) where only spikes in the longest burst were kept per lap and place field while the remaining spikes were deleted offline. The maximum inter-spike interval (ISI) was smaller than 20 ms. Single cell theta phase precession was eliminated with this process. **e**, Averaged decoding results during the first 2 detour laps after the spike deletion. Theta sequence can be clearly observed especially in lap 2. **f**, Distribution of theta sequence quadrant ratio after the spike deletion. Each dot represents one animal, direction, and detour session (n=20; Lap1, P=1.4x10-4; Lap2, P=0.0012; One side Wilcoxon signed rank test with positive quadrant ratio).

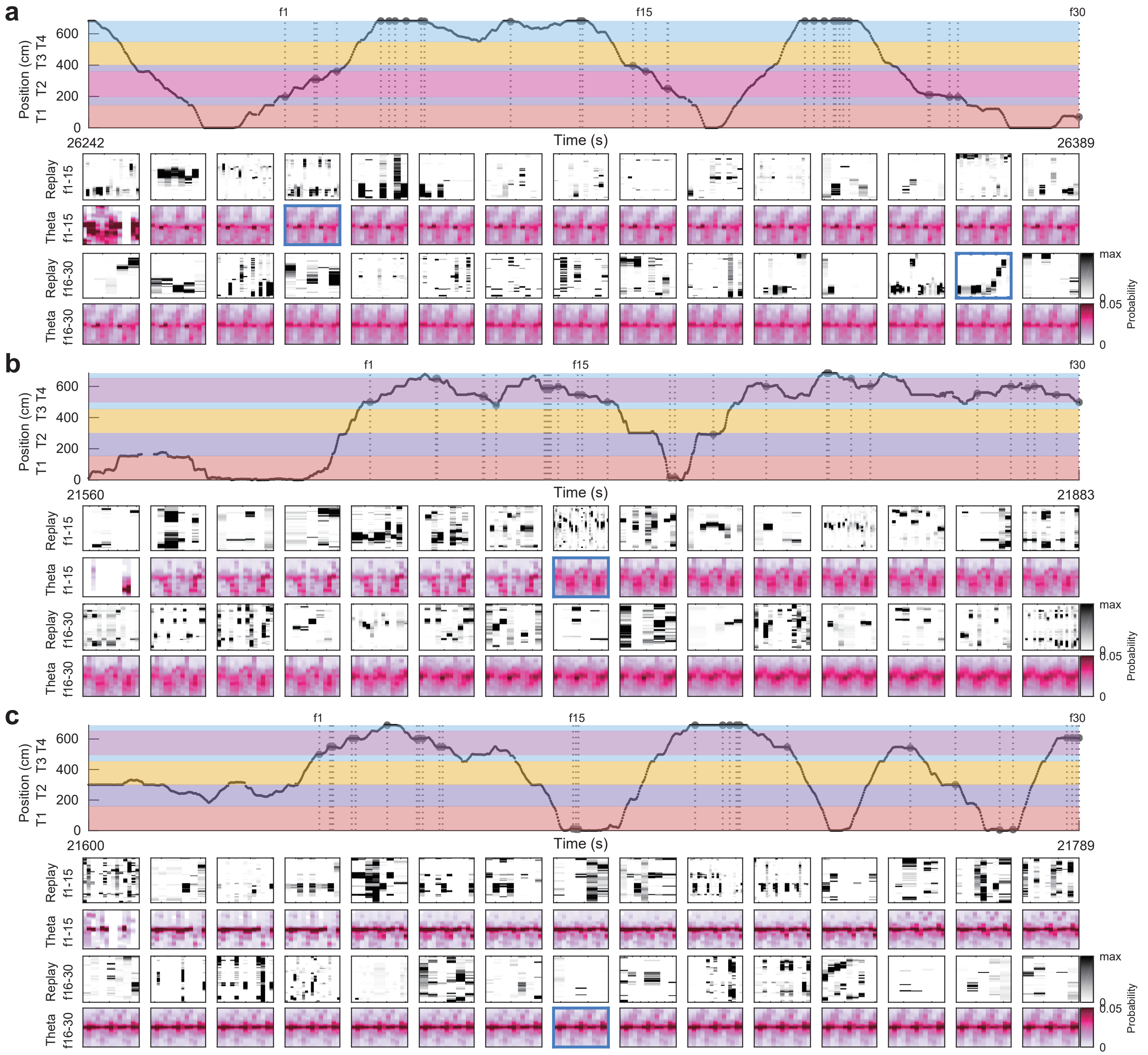

Supplementary Figure 5. Calculation of the time and lap of emergence of theta sequence and replay. a, Top panel, linearized position versus time for early laps when T2 was detoured. Tracks are color coded as in Fig. 1a. First 30 detected frames (f1-f30) are displayed following the animal's first moving step on the 150 cm detour segment for a given direction, marked as grey dashed lines and circles. Bottom panels (4 rows), detour decoding result for each detected frame (black colormap) and the theta sequence decoding result averaged across all the detour theta cycles before corresponding frame above (red colormap). The first detected significant replay (absolute weighted correlation >0.6; normalized maximum jump <0.4) is marked by blue and bold outline (if it happened within the first 30 frames). The theta decoding result where the theta cycles preceding that frame first exhibited significant positive quadrant ratios at population level (Wilcoxon signed rank with p value<0.01) is marked by blue and bold outline. **b-c**, Two other examples similar to (a) from different animals.

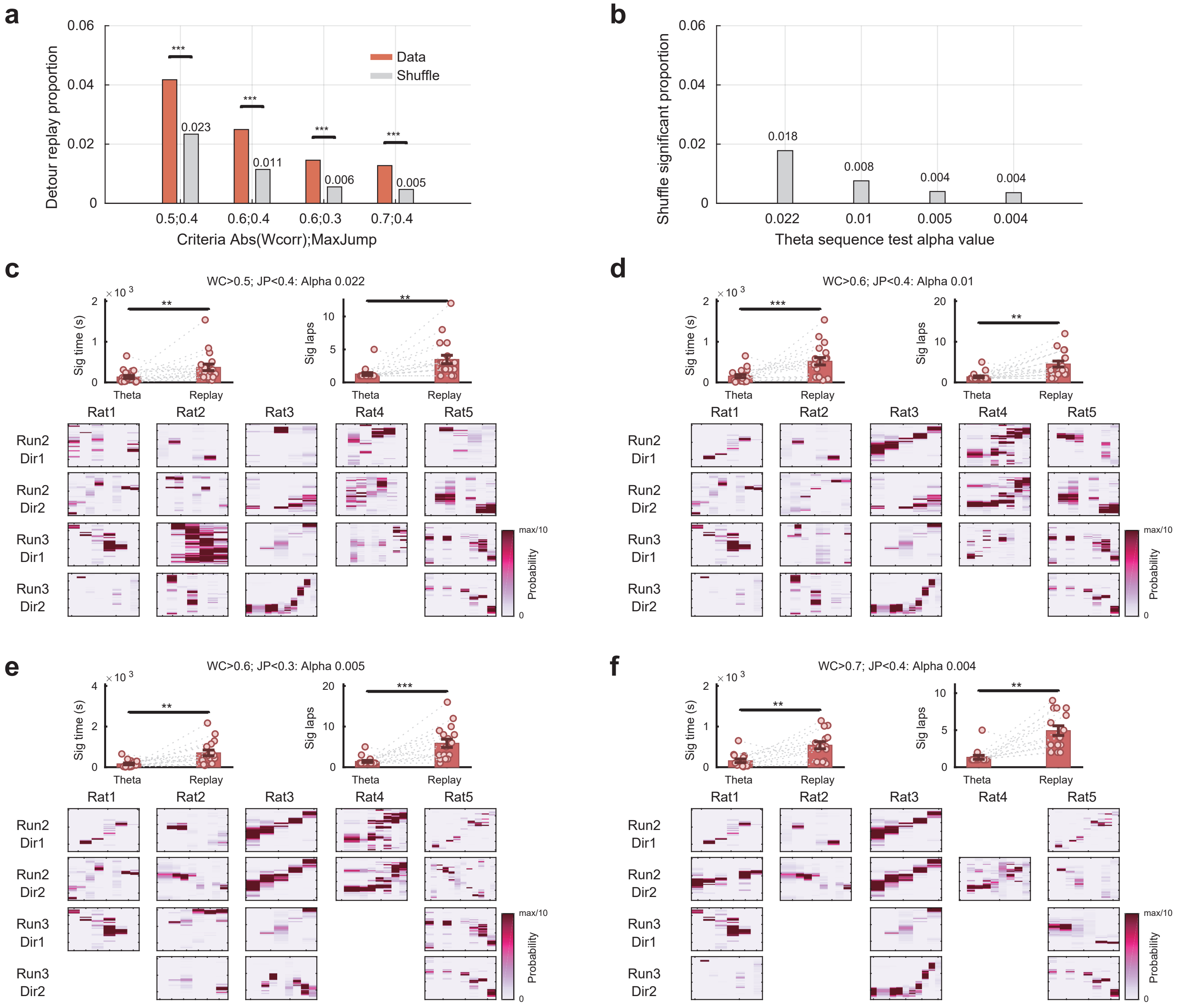

Supplementary Figure 6. Detection of replay and theta sequence with different significance criteria. a, Ratios of significant waking rest detour replay in real data and temporal shuffle control using a range of significance criteria. Values on the x-axis (e.g., 0.5; 0.4) represent absolute weighted correlation (e.g., >0.5) and normalized maximum jump (e.g., <0.4). Significant ratios from shuffle datasets were used as false positive rates of replay detection. The difference between data and shuffle was tested using Z-test for 2 proportions. **b**, Ratios of significant theta cycle subpopulations in temporal shuffle control with different alpha values for Wilcoxon signed rank test. Each time, 1,000 samples were drawn from the temporal shuffled theta cycles to form a subpopulation, and a Wilcoxon signed rank test was conducted to check if the subpopulation had significantly positive quadrant ratio. This procedure was repeated 1,000 times to get the significant ratio of subpopulations. **c-f**, Detour replays were detected using different significance criteria indicated by panels' titles (WC: absolute weighted correlation, JP: normalized maximum jump). The theta sequences were detected with corresponding false positive rate (indicated by alpha level) based on data displayed in (a). Significant time and lap for detour replays were compared with those for theta sequences (Top row). The first detected detour replay was plotted for each animal at Run2 direction 1, Run2 direction 2, Run3 direction 1, and Run3 direction 2. Empty panels signify that no replay was detected based on the significance criteria. In (c-f) data are displayed as mean±s.e.m. with each dot representing one animal, one direction, and one detour session (n=20). ***P<0.001, **P<0.01.

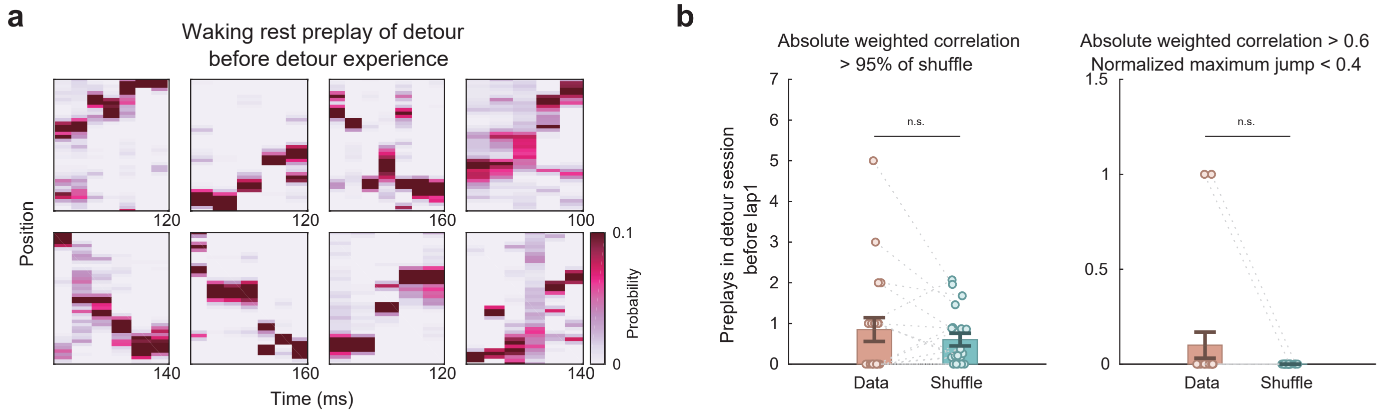

Supplementary Figure 7. Waking rest detour preplay can be detected when there are adequate waking rest frames. a, Examples of waking rest preplay of detour experience. Most examples were from waking rests during pre-detour sessions. **b**, Number of significant waking rest detour preplay during the detour session but before the animals first encounter of detour experience (animals entered detour from the other adjacent tracks). The waking rest detour preplay was detected as absolute weighted correlation larger than 95% of the shuffle (Left) or absolute weighted correlation larger than 0.6 and normalized maximum jump less than 0.4 (Right). Limited cases were detected and their number was not significantly higher than the shuffle control due to limited samples of rests. In (b), data are displayed as mean±s.e.m. with each dot representing one animal, one direction, and one detour session (n=20). n.s.=not significant.

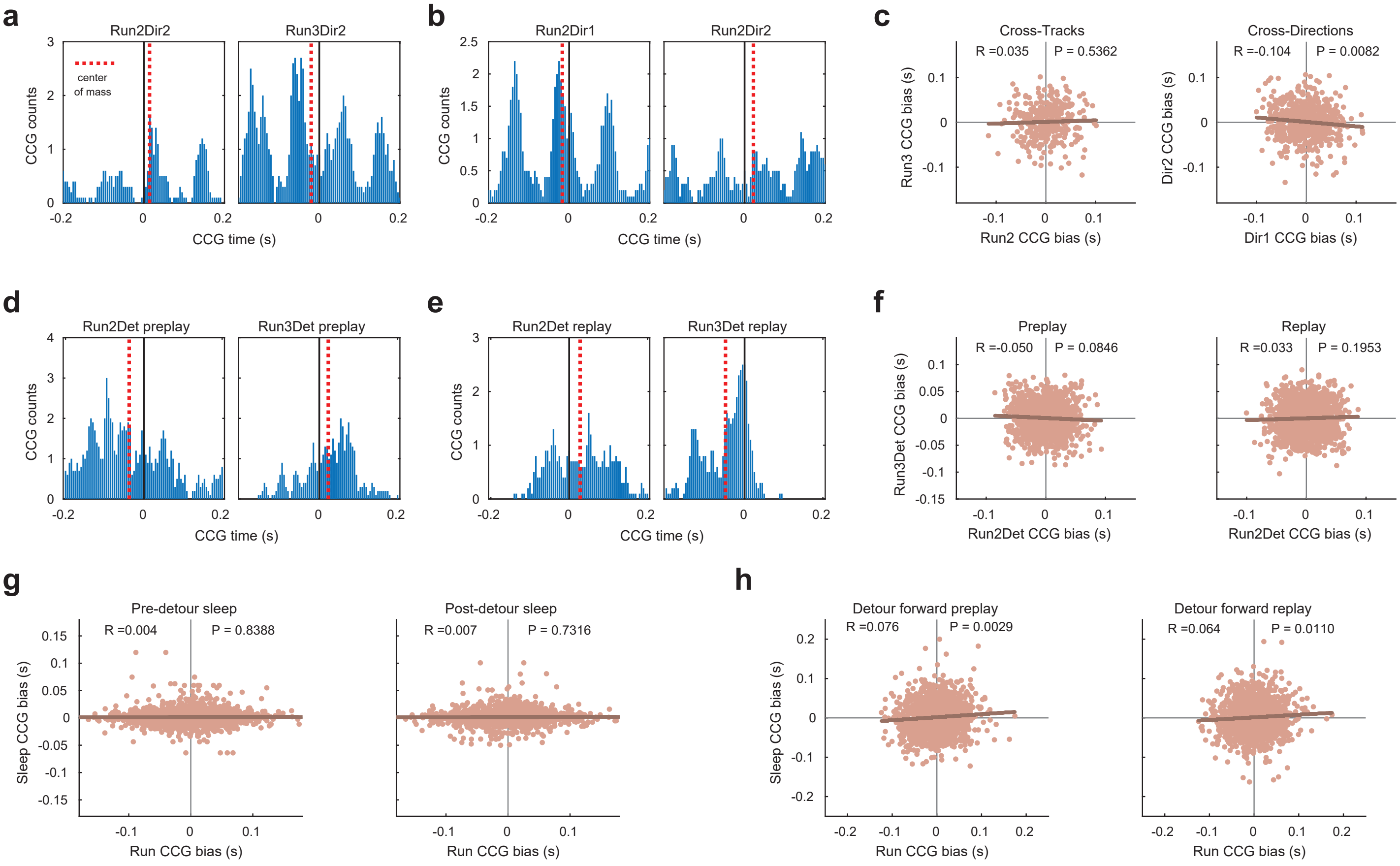

Supplementary Figure 8. Same cell-pair expresses different CCG patterns within detour run sessions and sleep sessions. a, Example cell-pair exhibiting different CCG temporal structures during two detour runs. **b**, Example cell-pair exhibiting different CCG temporal structures across two run directions during the same detour run. **c**, CCG temporal biases were uncorrelated between two detour runs (left) and had negative correlation between two run directions during detour (right). **d**, Example cell-pair exhibiting different CCG temporal structure based on frames which are preplay of run2 detour or preplay of run3 detour. **e**, Example cell-pair exhibiting different CCG temporal structure based on frames which are replay of run2 detour or replay of run3 detour. **f**, CCG temporal biases were uncorrelated between two detour preplays (left) or replays (right). **g**, CCG temporal biases were uncorrelated between detour runs and the entire pre- (left) or post-detour sleep (right). **h**, CCG temporal biases were correlated between detour run and sleep if frames were selected as significant forward detour preplays (left) or detour replays (right). Each dot represents one cell-pair. Results were pooled across animals.

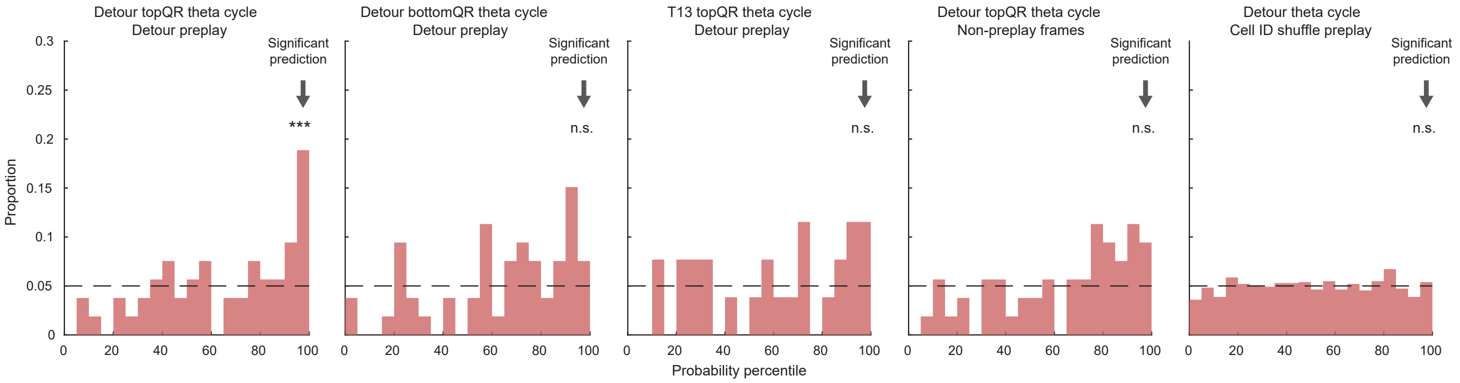

Supplementary Figure 9. Control for Markov model prediction. Prediction probability vs. shuffle sequence percentile with different configurations. From left to right: Transition matrix from forward detour preplay to predict early detour theta cycles with top 5% quadrant ratio; Transition matrix from forward detour preplay to predict early detour theta cycles with bottom 5% quadrant ratio; Transition matrix from forward detour preplay to predict early T1&T3 theta cycles with 5% quadrant ratio; Transition matrix from the same number of frames with low absolute weighted correlation to predict early detour theta cycles with 5% quadrant ratio; Transition matrix from forward detour preplays after a cell ID shuffle. Bar plots display mean±s.e.m. ***P<0.001, n.s.=not significant.

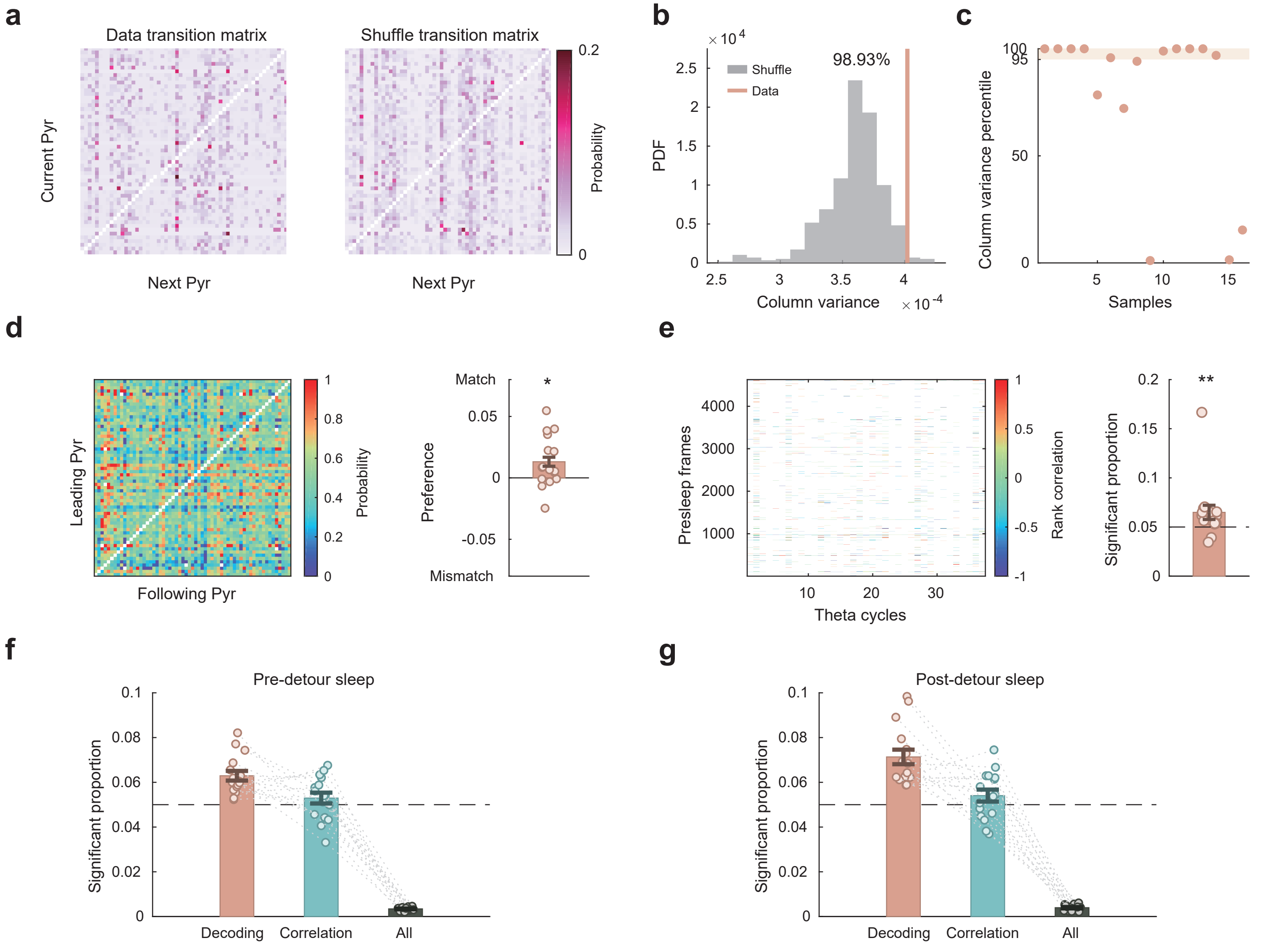

Supplementary Figure 10. **Order-related correlation between early theta cycle activities and pre-detour sleep activities. a**, Example Markov model transition matrix computed from forward detour prelays (left), and the Markov transition matrix computed from the same population of frames but with independent within-frame shuffle of sequence order (right). The within-frame sequence order shuffle disrupted the relative order structures of cell-pairs and made the transition matrix display stronger continuous column structure (i.e., stripes), which were dominated by neuronal firing rates. **b**, The contribution of sequence order structure to the Markov transition matrix was quantified as the variance along columns in the transition matrix. Independent within-frame sequence order shuffle reduced the variance along columns. The column variance of the example transition matrix was among 98.93% percentile compared with sequence shuffles. **c**, Distribution of column variance percentile against sequence order shuffle across animals, directions, and detour sessions (Rat4 was excluded from sleep-related analysis due to low cell and frame counts). **d**, Example of pairwise spike sequence probability matrix computed from forward detour preplays. Each pixel represents the probability that the row cell fires before the column cell (measured by center of mass of spike times) if they are both active in a frame. This measure was not dependent on firing rates (Left). Based on the pairwise spike sequence probability matrix, we measured the probability of pairwise activities during early detour laps. For each animal, direction, and detour session, we got an average probability across all cell pairs, and compared the results to the 0.5 chance level (Right). Positive results indicated that the probability was higher than 0.5 chance level and the relative order in early theta cycles was compatible with forward detour preplay. **e**, Example showing the rank order correlation of spike sequence order between early laps theta cycles with not less than 5 active cells (x-axis) and pre-detour sleep frames (y-axis). If the pair had less than 5 common active cells, the result was not shown given there were not enough independent permutations to compute a significant measure (Left). Across all the pairs with not less than 5 common active cells, the ratio of pairs with significant correlation (>95% of sequence shuffle) was computed and compared with 5% chance level (Right). **f**, Proportions of significant detour preplay detected by Bayesian decoding, rank order correlation, or significant by both methods. **g**, Proportions of significant detour replay detected by Bayesian decoding, rank order correlation, or significant by both methods. Bar plots in (d)-(g) are displayed as mean±s.e.m. with each dot representing one animal, one direction, and one detour session (n=16). **P<0.01, *P<0.05.

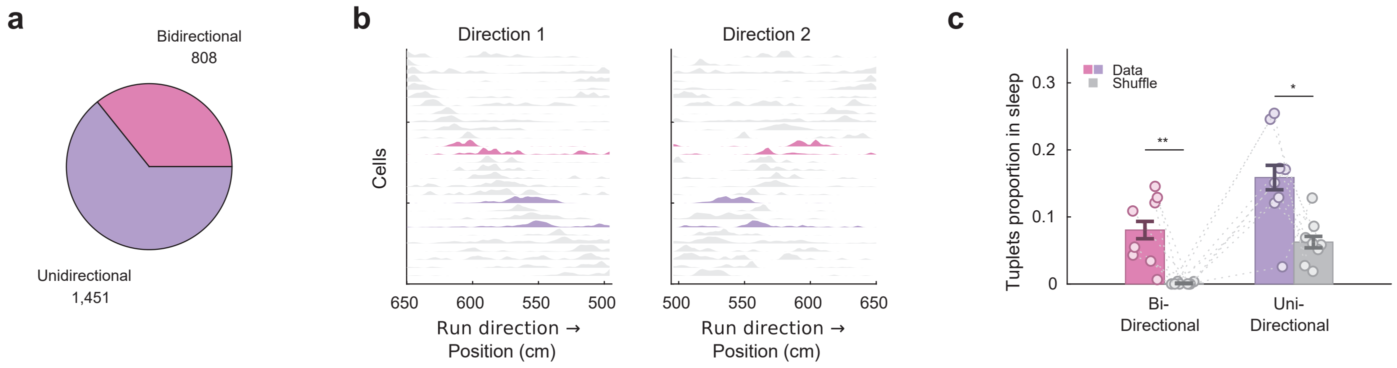

Supplementary Figure 11. Bidirectional 2-neuron tuplets during sleep correlated with bidirectional pairwise sequential structure during run. a, Distribution of 2-neuron tuplets detected in pre-detour sleep. Among 2,259 tuplets, 808 of them were bidirectional meaning the mirror copy was also detected as a tuplet. **b**, Example showing place map sequence of the detour segment across two run directions. Only cells which were active in both directions were plotted. Cells were sorted based on their peak firing rate locations in direction 1. Note the x-axis in direction 1 was reversed and in both displayed directions animals were running from left to right. A bidirectional cell pair was marked in red with flipped sequence order across directions. A unidirectional cell pair was marked in purple with preserved sequence order across directions. **c**, Bidirectional and unidirectional pairwise sequences during the detour run were significantly pulled from bidirectional and unidirectional tuplets detected in pre-detour sleep (Bidirectional data vs. shuffle P=0.0078; Unidirectional data vs. shuffle P=0.0391; Wilcoxon signed rank test). Each dot represents one animal and detour session (n=8).

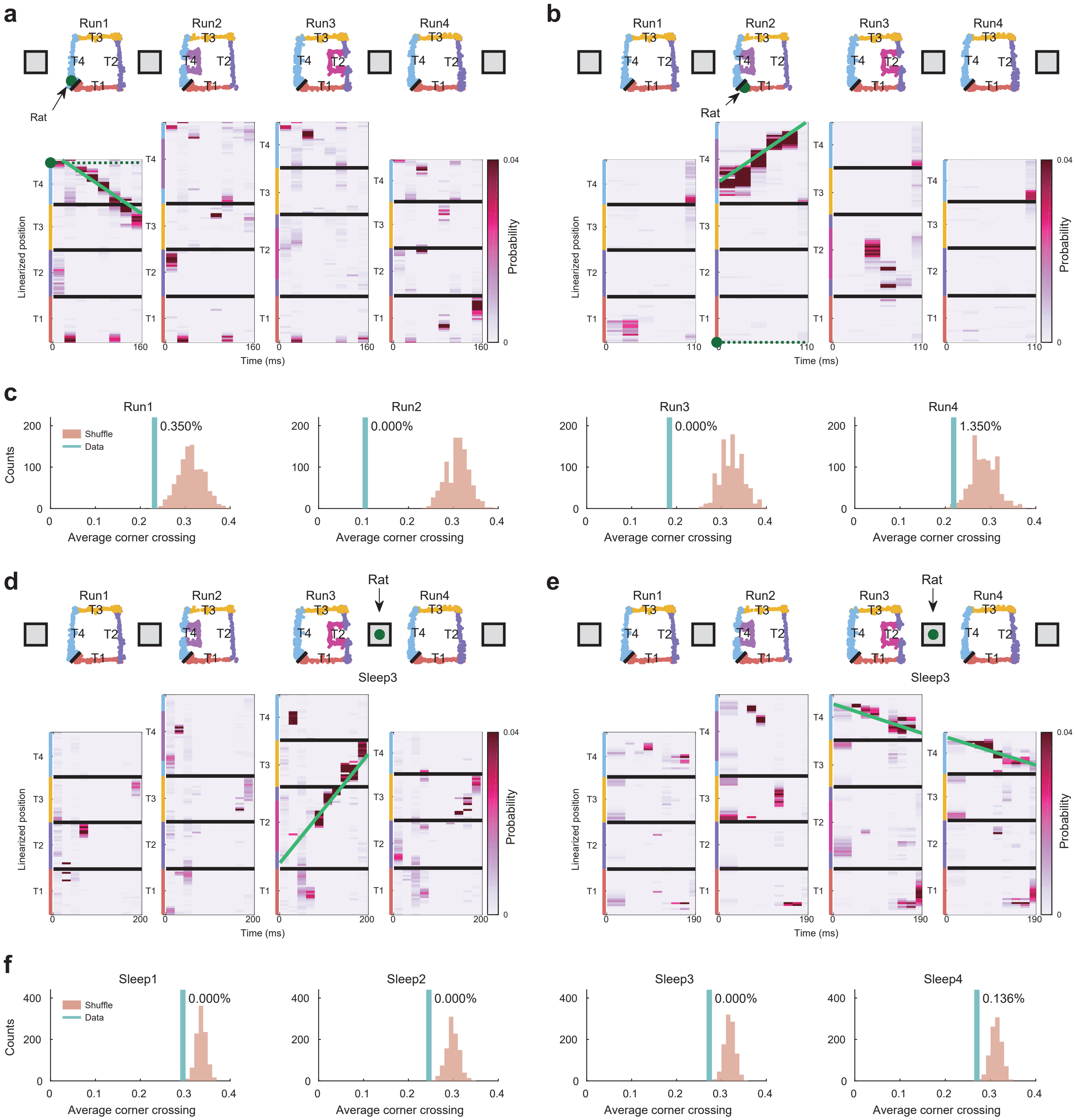

Supplementary Figure 12. Waking rest and sleep p/replay near maze corners. a-b, Examples of waking rest replays crossing maze corner (a) or confined within the detour track (b). Top row shows run sessions and tracks with animal actual position marked as dark green dot on the track. Bottom row shows decoding probability computed separately within each session but subsequently concatenated across tracks. Dark green dashed line marks the animal's actual position. Thick black lines mark maze corners. For significant p/replay, the best linear fit of the decoded trajectory was plotted as a continuous green line. Number of maze corner crossings were computed based on the linear fit. **c**, Average number of maze corner crossings compared with shuffle dataset across the four run sessions. The numbers of maze corner crossings were significantly lower than shuffle during waking rest for all four run sessions. **d-e**, Examples of sleep replay crossing maze corner (d) or confined within the decoded track (e). Sleep sessions were indicated by animals resting in the sleep box across run sessions. **f**, Average number of maze corner crossings were significantly lower than shuffle in all sleep sessions.

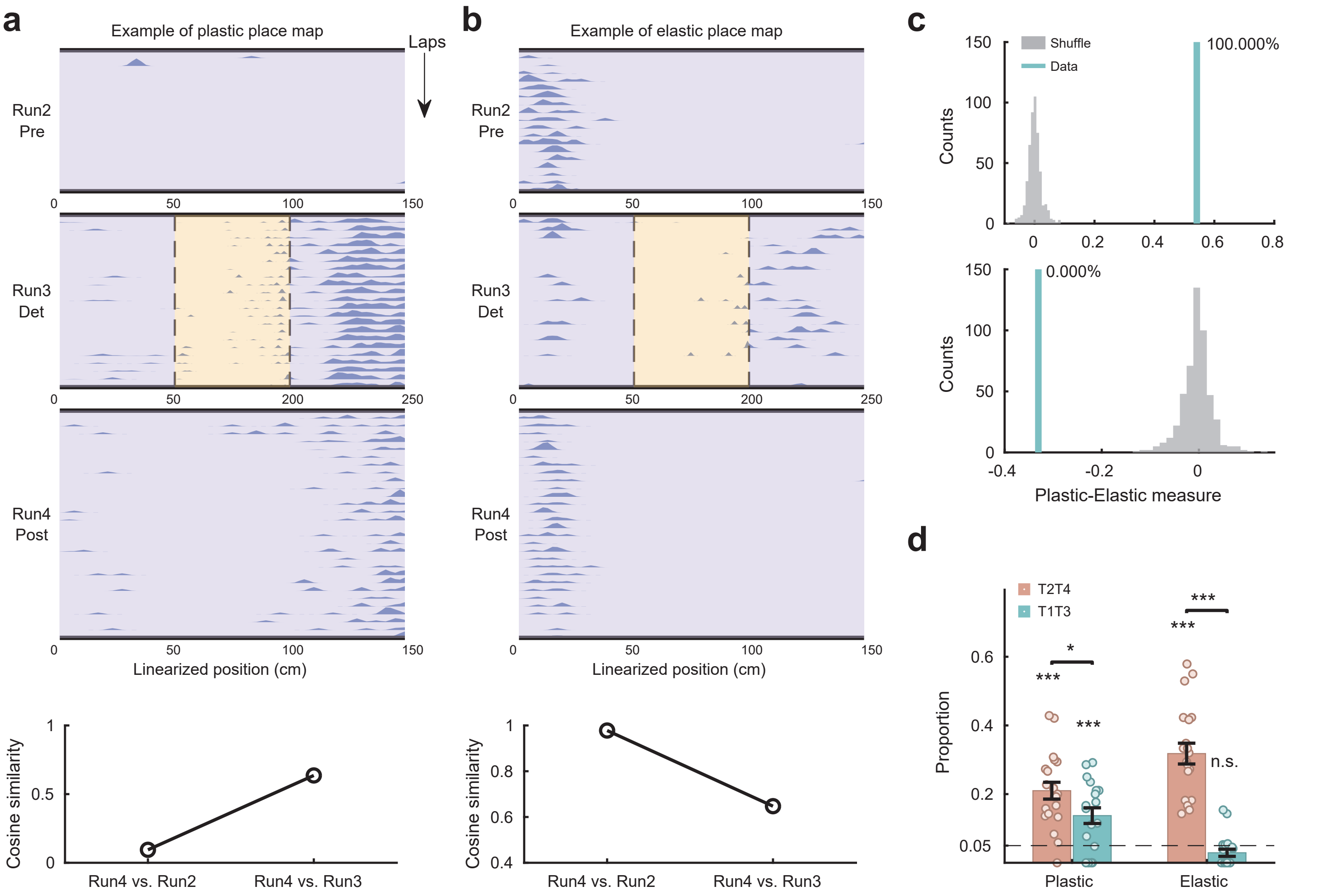

Supplementary Figure 13. Detour-related plastic and elastic place maps. a, Example of one plastic place map. Top, lap by lap place maps were plotted in pre-detour, detour and post-detour sessions. The 1.5 m detour segment during the detour session is displayed in yellow and was compressed to fit in the 0.5 m range to allow for direct cross session comparison. Bottom, place map cosine similarity across sessions. The place map similarity was computed based on the two 50 cm stationary segments of detoured tracks as there was no explicit correspondence for the middle segment. **b**, Example of one elastic place map. Configuration is as in (a). **c**, Top, plastic-elastic measure of example from (a) compared with lap shuffle. The plastic-elastic measure was defined as post-detour vs. detour similarity minus post-detour vs. pre-detour similarity. The plastic-elastic measure of the data is plotted as a blue line and compared against surrogate datasets where laps were randomly shuffled 500 times, and the measures were computed. Data exceeding 95% percentile of shuffle was defined as a plastic place map. Bottom, plastic-elastic measure of example from (b) compared with lap shuffle. Data less than 5% percentile of shuffle was defined as an elastic place map. **d**, Ratio of detour related plastic and elastic place maps for T2-T4 and T1-T3. For T1 and T3, virtual detour sessions were assigned as described in methods. Dashed line marks 5% chancel level. Detour tracks had significant plastic and elastic place maps compared with chance level (Plastic P<10-5; Elastic, P<10-7, paired t-tests). T1 and T3 only had significant plastic place maps compared with chance level (Plastic P=5.7×10-4; Elastic, P=0.9704, paired t-tests). Detour tracks had significantly higher plastic and elastic place maps ratio than T1-T3 (Plastic P=0.0117; Elastic, P=6.7×10-9, paired t-tests). Each dot represents one animal, direction, and track (n=20).

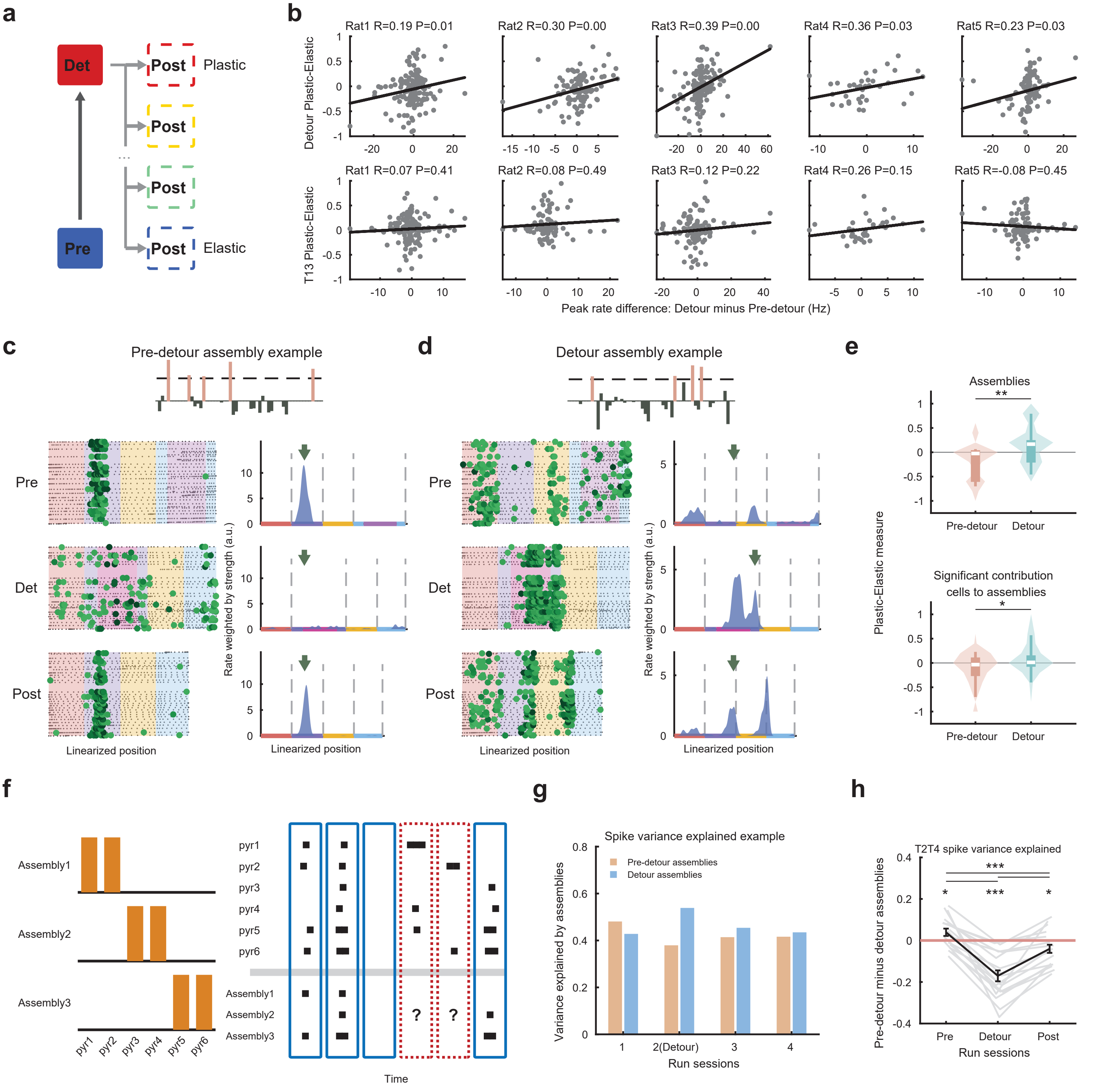

Supplementary Figure 14. Post-detour plasticity predicted by detour activities. **a**, Cartoon illustrating plastic-elastic measures. The measure =1 (plastic) if post-detour tuning curve was similar to detour, and =-1 (elastic) if post-detour tuning curve was similar to pre-detour. **b**, Cell's plastic-elastic measure was correlated with firing rate change from pre-detour to detour session on detoured tracks (top row), but not on non-detoured tracks (bottom row). **c**, Example pre-detour assembly with its activities and spatial tunings across sessions. **d**, Example detour assembly with its activities and spatial tuning across sessions. **e**, Detour assemblies had more plastic spatial tuning than pre-detour assemblies. Cells significantly contributing to detour assemblies had more plastic spatial tuning than those contributing to pre-detour assemblies. **f**, Cartoon illustrating cell activity explained by significant assemblies. Blue rectangle represents spike patterns that could be, while red dashed rectangle spike patterns that could not be, fully explained by assemblies. **g**, Example showing across run sessions the variance of spike activity explained by pre-detour and detour assemblies. **h**, Difference of spike variance explained by pre-detour and detour assemblies during pre-detour, detour, and post-detour sessions. In (h) data displayed as mean±s.e.m. with each line representing one animal, direction, and detour session (n=20). ***P<0.001, **P<0.01, *P<0.05.

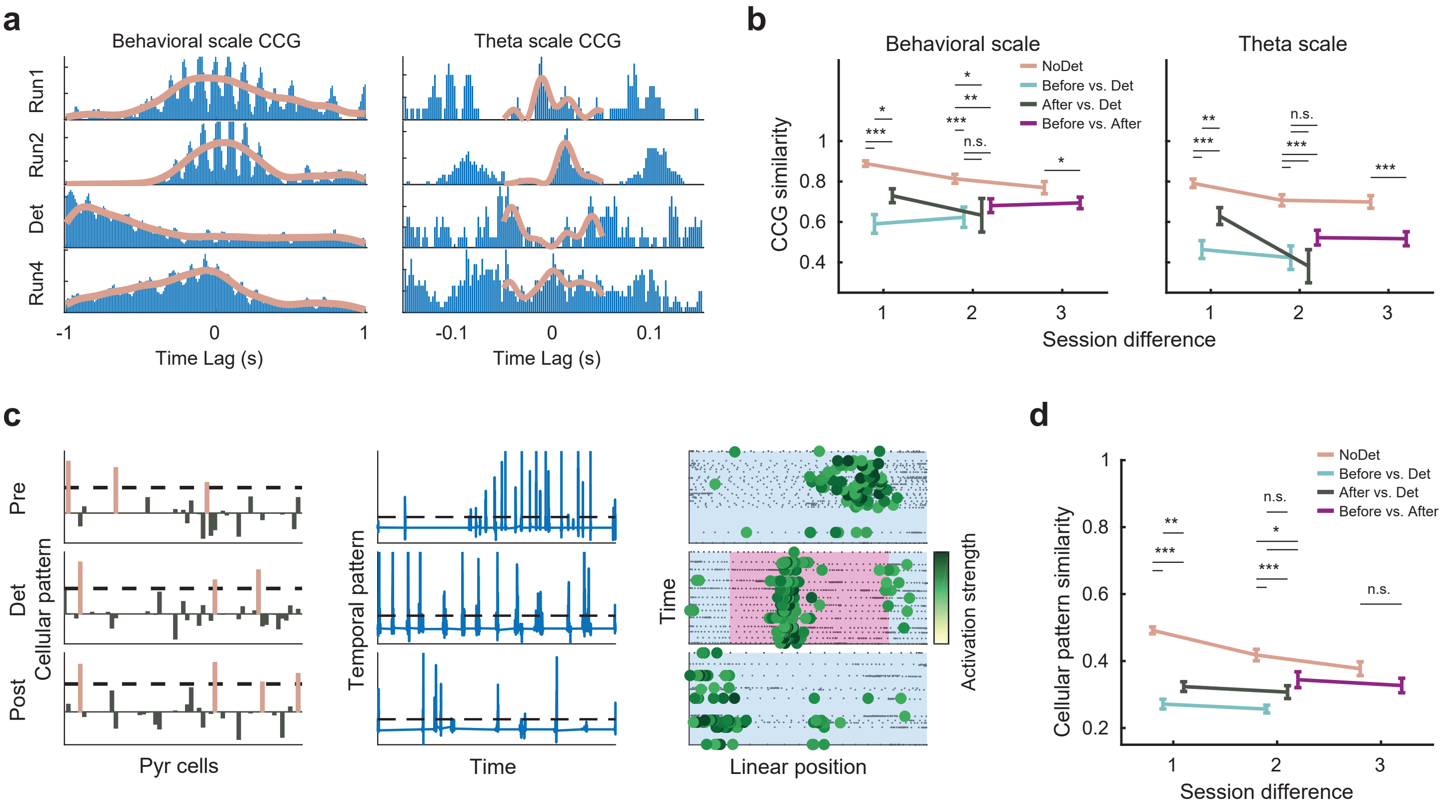

Supplementary Figure 15. Detour-induced plasticity measured at pairwise and ensemble level. a, CCG examples from one cell pair across run sessions at behavioral and theta time scales. The CCGs were computed based on spikes on the entire track rather than stationary segments. Note that the detour experience impacted on the CCG pattern, which was not fully restored in the reversal run. The orange lines mark the low pass-filtered CCGs at behavioral time scale (<0.5 Hz, from -1s to 1s) and theta time scale (<30 Hz, from -50 ms to 50 ms) which were used to compute CCG similarity across sessions. **b**, CCG similarity measured at behavioral time scale (left) and theta time scale (right) grouped by relations to detour and plotted against session difference. Detoured tracks experienced larger CCG drift compared with non-detoured tracks throughout the experiment. **c**, Example of cell assemblies detected on the detoured track in pre-detour, detour and post-detour sessions. Left panels show cellular pattern of cell assemblies with significantly contributing cells marked in orange. Middle panels show temporal pattern of cell assemblies with removed single cell contribu-tion. Right panels show activation time of cell assemblies during maze run. **d**, Similarity of assemblies was measured as the highest correlation of cellular patterns of detected cell assemblies across sessions. Detoured tracks experienced a larger assembly change compared with non-detoured tracks throughout the experiment.

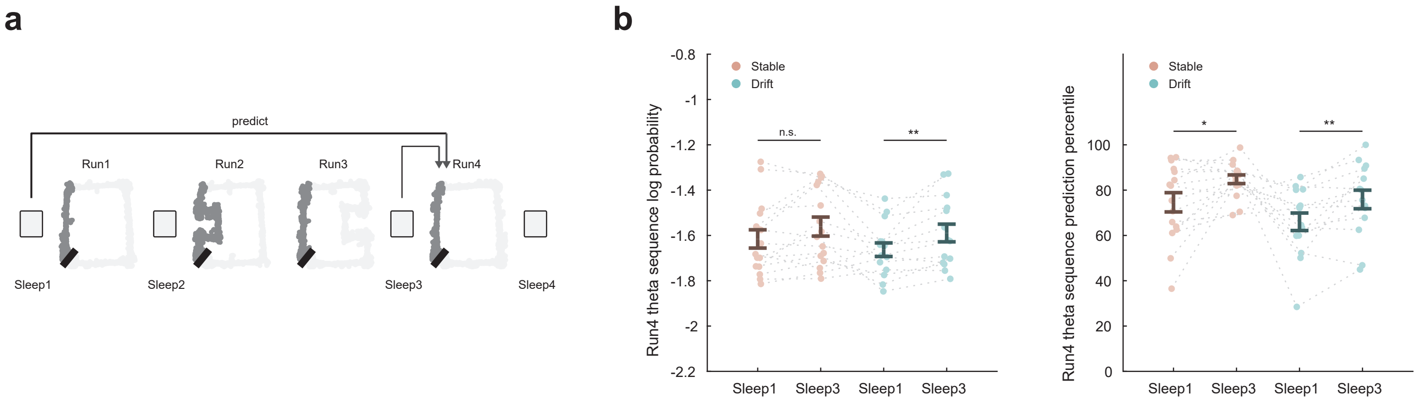

Supplementary Figure 16. Sleep predicted future theta sequence during the reversal run. a, Using Sleep 1 or Sleep 3 to predict drift of stable theta sequence during reversal Run4. **b**, (Left) Run4 normalized probabilities for stable and drift theta sequences predicted from Sleep1 and Sleep3. Each dot represents prediction probability averaged across theta cycles for each animal, direction, and detoured track. (Right) Run4 stable and drift theta sequences probability percentiles compared against shuffle sequences predicted by Sleep1 and Sleep3. In (b) data displayed as mean±s.e.m. with each line representing one animal and direction (n=16). **P<0.01, *P<0.05, n.s.=not significant.

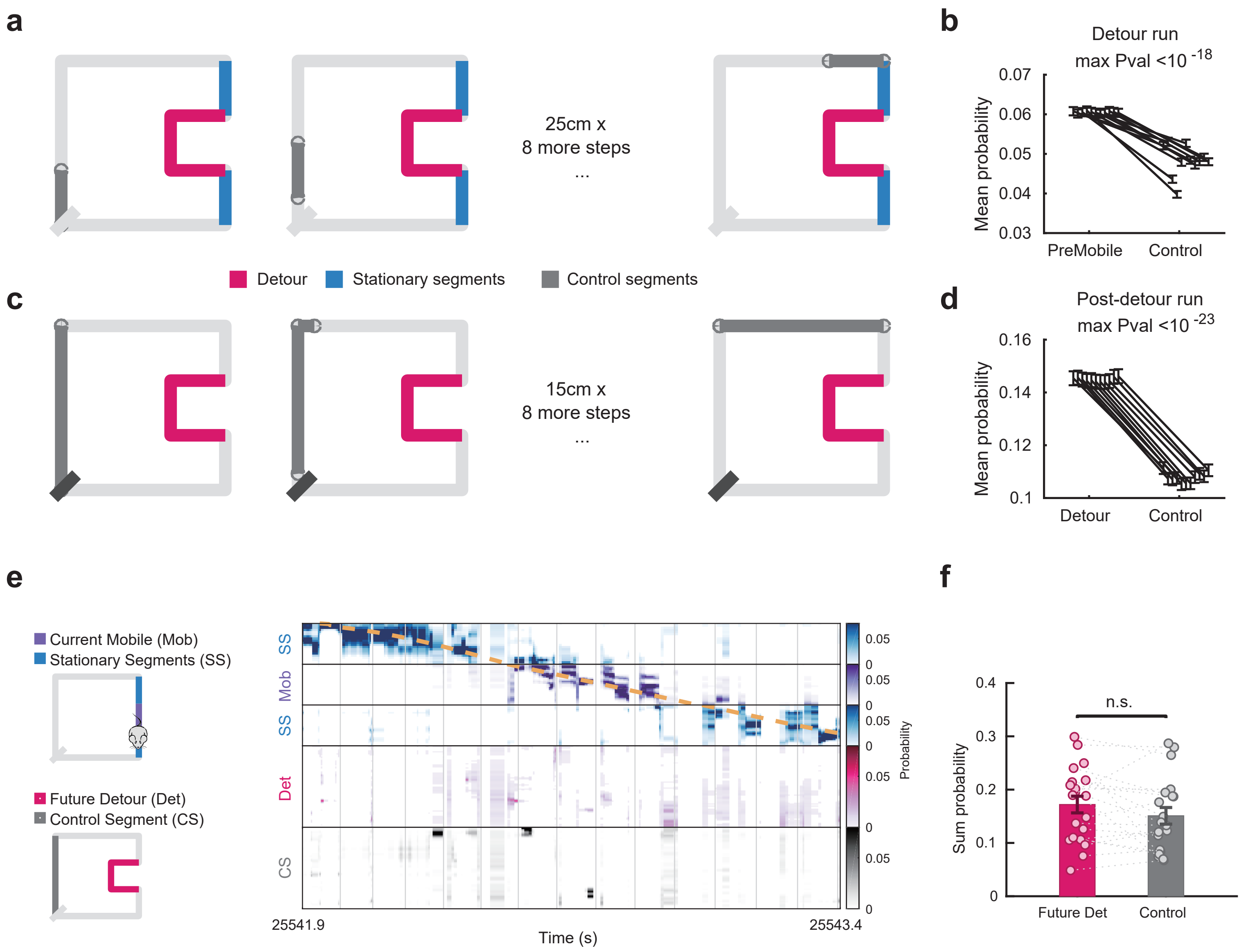

Supplementary Figure 17. Flickering controlled by sliding control segments and pre-detour decoding. a, Sliding control segments compared with pre-detour mobile segments of a detoured track using Bayesian decoding during a detour run. 50-cm control segments sliding from the end of the opposite track to the start of the adjacent track in steps of 25 cm. For detoured T2, control segments sled from T4 to T3. For detoured track 4, control segments sled from T2 to T1. **b**, Decoding probability for pre-detour mobile and sliding control segments. Each line represents one control segment. The largest P value was shown (Wilcoxon signed rank test). **c**, Sliding control segments compared with the detour segment during post-detour run decoding. The stepping size of sliding was 15 cm. **d**, Decoding probability between detour and controls using different control segments. **e**, Decoding configuration and example during pre-detour run displaying probabilities for the current mobile segment (later removed), two stationary linear segments, future detour segment and control segment. **f**, Decoding probability of future detour was not significantly higher than of control segment during pre-detour run. Each dot represents one animal, direction, and pre-detour session (n=20; P=0.0793; Wilcoxon signed rank test).

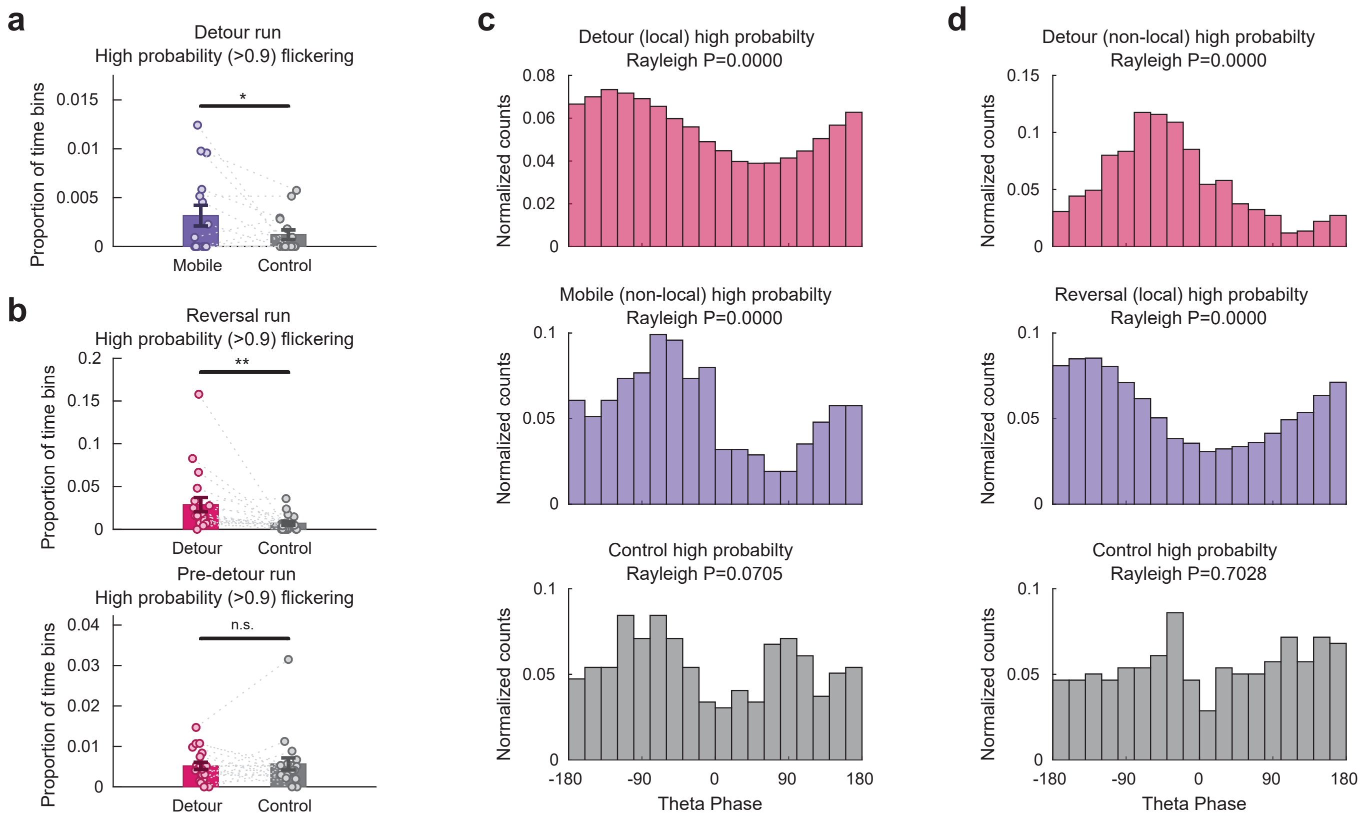

Supplementary Figure 18. Flickering measured by high probability representation. a, During detour run, the ratio of time bins with more than 3 active cells and decoding probability of pre-detour mobile segment higher than 0.9 were compared against ratio of time bins with decoding probability of control segments higher than 0.9. **b**, Top panel: during reversal run, the ratio of time bins with detour decoding probability higher than 0.9 compared against control decoding probability higher than 0.9. Bottom panel: same measure as top panel during the pre-detour session. **c**, During the detour run, the theta phase distribution of time bins with strong representation (probability > 0.9) of detour segment (top), pre-detour mobile segment (middle), and control segment (bottom). The distributions are tested with the Rayleigh test for uniformity. **d**, During the reversal run, the theta phase distribution of time bins with strong representation (probability > 0.9) of detour segment (top), reversal segment (middle), and control segment (bottom). Note that in (c, d) the local representation is stronger near theta troughs while the alternative non-local context is represented stronger near theta peaks. Control segments have relatively uniform distribution. Bar plots in (a, b) are displayed as mean±s.e.m. with each dot representing one animal, direction, and detour session (n=20). **P<0.01, *P<0.05, n.s.=not significant.
